# Neurochemical Plasticity of nNOS-, VIP- and CART-Immunoreactive Neurons Following Prolonged Acetylsalicylic Acid Supplementation in the Porcine Jejunum

**DOI:** 10.3390/ijms21062157

**Published:** 2020-03-20

**Authors:** Dominika Rząp, Marta Czajkowska, Jarosław Całka

**Affiliations:** Department of Clinical Physiology, Faculty of Veterinary Medicine, University of Warmia and Mazury in Olsztyn, Oczapowskiego Str. 13, 10-718 Olsztyn, Poland; marta.czajkowska@uwm.edu.pl (M.C.); calkaj@uwm.edu.pl (J.C.)

**Keywords:** neuroprotection, aspirin, ENS, peripheral nervous system, inflammation, pig

## Abstract

Aspirin, also known as acetylsalicylic acid (ASA), is a commonly used anti-inflammatory drug that has analgesic and antipyretic properties. The side effects are well known, however, knowledge concerning its influence on gastric and intestinal innervation is limited. The enteric nervous system (ENS) innervates the whole gastrointestinal tract (GIT) and is comprised of more than one hundred million neurons. The capacity of neurons to adapt to microenvironmental influences, termed as an enteric neuronal plasticity, is an essential adaptive response to various pathological stimuli. Therefore, the goal of the present study was to determine the influence of prolonged ASA supplementation on the immunolocalization of neuronal nitric oxide synthase (nNOS), vasoactive intestinal peptide (VIP) and cocaine- and amphetamine- regulated transcript peptide (CART) in the porcine jejunum. The experiment was performed on 8 Pietrain × Duroc immature gilts. Using routine double-labelling immunofluorescence, we revealed that the ENS nerve cells underwent adaptive changes in response to the induced inflammation, which was manifested by upregulated or downregulated expression of the studied neurotransmitters. Our results suggest the participation of nNOS, VIP and CART in the development of inflammation and may form the basis for further neuro-gastroenterological research.

## 1. Introduction

The digestive system plays an essential role in the supply of nutrients, digestion and absorption and has a decisive influence on the entire organism. Moreover, unlike other internal organs, the alimentary tract is exposed to different stimuli from the external environment in the form of ingested food and drugs. As a consequence, the stomach and intestine have developed a large variety of adaptations to ensure the appropriate mixing and transit of intestinal contents during digestion, absorption and excretion [1]. The digestive system is known to be regulated by extrinsic innervation (sympathetic, parasympathetic, sensory ganglionic neurons), and by intrinsic innervation—intramural neurons positioned in the wall of the entire gastrointestinal tract (from the esophagus to the anus) forming the enteric nervous system (ENS) [2,3]. The ENS, also called the second brain, consists of a mesh-like system of neurons and is capable of acting independently of the sympathetic and parasympathetic nervous systems [4]. The ENS, outside the central nervous system, is one of the main regulators of gastrointestinal (GI) function and contributes to tissue response to pathological conditions [5]. In the porcine jejunum, the ENS is comprised of the myenteric plexus (MP), situated between the longitudinal and circular layers of the external musculature layers and the inner (ISP) and outer (OSP) submucosal plexuses [6,7]. The MP primarily regulates normal gastric motility, while the OSP and ISP plexuses are mainly responsible for fluid secretion and resorption [8].

Aspirin (acetylsalicylic acid) is one of the most commonly used medications in the world and is classified as a non-steroidal anti-inflammatory drug (NSAID), which has anti-inflammatory, analgesic and antipyretic properties [9]. Additionally, it suppresses the normal functioning of platelets. According to Fuster and Sweeny [10], around 40,000 tons of aspirin are produced each year worldwide, while in the USA alone, more than 50 million people take 10 to 20 billion aspirin tablets regularly to prevent cardiovascular diseases. Aspirin is excellent in the prophylaxis of cardiovascular diseases. Low doses of acetylsalicylic acid are used in the prevention of stroke, ischemic heart disease, myocardial infarction and peripheral vascular disease [11]. Other benefits include pain relief (analgesia), treatment of the symptoms of various inflammatory conditions, reducing the risk of certain types of cancer, particularly colorectal cancer [10].

Acetylsalicylic acid (ASA) acts by irreversibly inhibiting cyclo-oxygenase enzyme (COX) in a non-selective way which leads to suppression of prostaglandins (PGs) and thromboxanes (TXAs) production [11]. Even low doses of aspirin cause superficial injury in the gastric epithelium and lead to incorrect ion flow characterized by increased H^+^ back diffusion. It is well known that COX has at least two different isoforms: the constitutive isoform (COX-1) and the inducible isoform (COX-2). COX-1 is present physiologically in the stomach, endothelium and kidney. Activation of COX-1 results in the production of prostacyclin, which has anti-thrombogenic and cytoprotective properties when released by the endothelium and gastric mucosa, respectively [12,13]. COX-2 is formed during the inflammatory process as a result of the action of endotoxins and proinflammatory cytokines and is responsible for the induction of prostaglandin’ production that causes pain and swelling [12,13,14,15]. Although the therapeutic benefits of using NSAIDs are well known, nonsteroidal anti-inflammatory drugs are associated with a variety of serious gastrointestinal side effects (ulceration, hemorrhage, perforation) and lead to damage throughout the gastrointestinal tract (GIT) [16].

The ENS is characterized as high plasticity due to its response to a variety of pathological stimuli [17]. This plasticity can be attributed to the ability of enteric neurons to change their chemical code. In each neuron, synthesis, storage and final release of the main neurotransmitter and other neuromodulators or cotransmitters (mainly neuropeptides) occurs. Adaptation to the changing environmental conditions includes changing in the chemical coding of neurons by enhanced expression of some neurotransmitters and neuromodulators and decreased of others [2,18,19]. Vasoactive intestinal peptide (VIP) and nitric oxide synthase (NOS) are well-known and widely described biologically active substances found in the ENS [2,3,20,21]. Furthermore, these neurotransmitters are involved in gut neuronal defense system [22]. Cocaine- and amphetamine-regulated transcript (CART) is relatively recently discovered peptide [23]. CART is implicated in a variety of physiological processes, including gastric acid secretion, stress response [24] and neuroprotection [23,25,26].

The goal of the present study was to determine the influence of high doses of acetylsalicylic acid (ASA) on the neurons in the enteric nervous system (ENS) of the porcine jejunum. It should be noted that selection of the drug active substance and animal species model were not accidental. Much attention is being devoted to utilizing animal models to study the pathogenesis of gastrointestinal disorders in response to various pathological stimuli. Swine are considered one of the main types of animals in biomedical research. Due to its omnivorous gut morphology and physiology, which are more comparable to those in humans than in rodents, pigs are an alternative model of human gastrointestinal diseases [27,28]. Moreover, this study seems to be especially justified due to chosen drug active substance, because nowadays aspirin is worldwide used as a medicine.

## 2. Results

**Figure 1:**
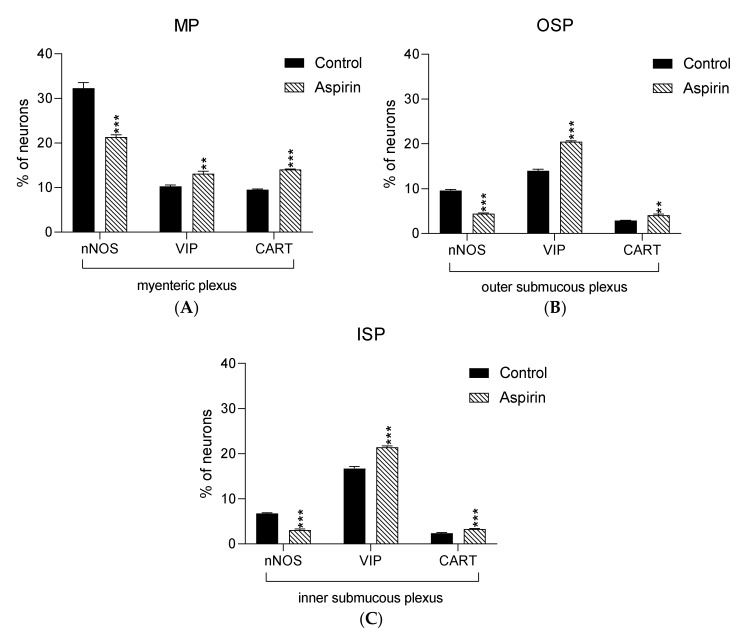
Graphs presenting percentage of nNOS-IR, VIP-IR and CART-IR neurons in the myenteric plexus (**A**) the outer submucous plexus (**B**) and the inner submucous plexus (**C**) within jejunum in the control (black bar) and experimental group (linked bar). ** *p* < 0.01, *** *p* < 0.001 indicates differences in expression of particular studied substance in comparison to control pigs.

**Figure 2:**
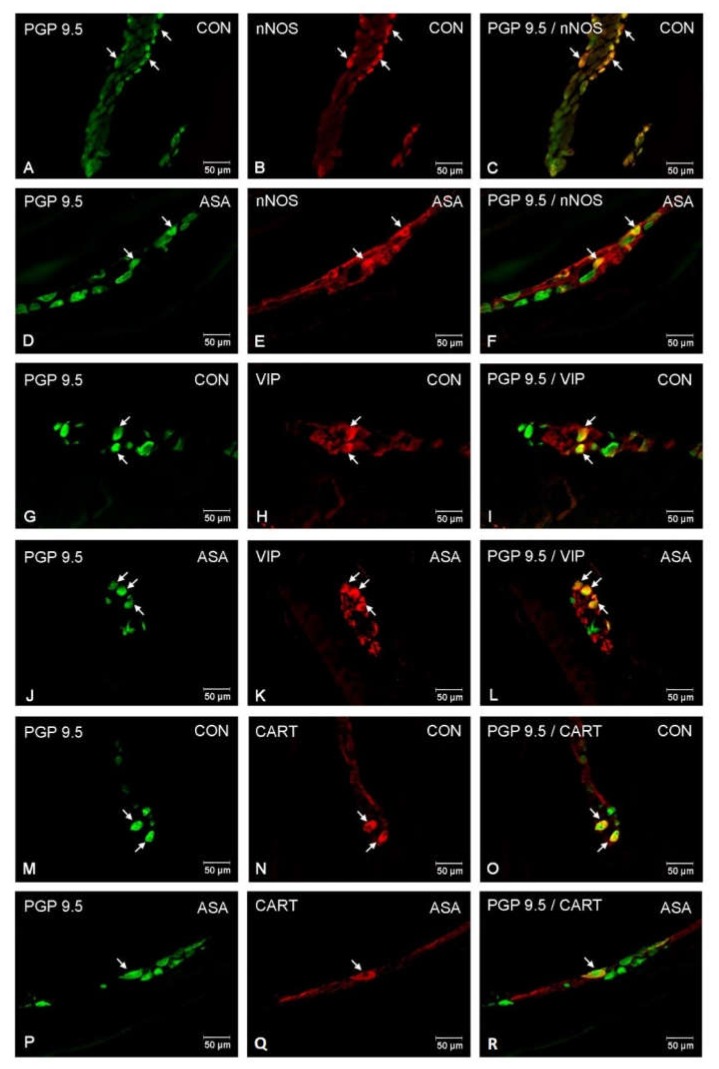
Neuron bodies within myenteric plexuses of porcine jejunum containing the protein gene-product 9.5 (PGP 9.5) (used as pan neuronal marker), neuronal nitric oxide synthase (nNOS), vasoactive intestinal peptide (VIP) and cocaine- and amphetamine- regulated transcript peptide (CART) under physiological condition (CON) (**A**–**C**,**G**–**I**,**M**–**O**) and after aspirin supplementation (ASA) (**D**–**F**,**J**–**L**,**P**–**R**). The right column of the pictures (**C**,**F**,**I**,**L**,**O**,**R**) has been created by digital superimposition of two colour channels, and colocalization of both antigens in the studied neuron bodies was indicated with arrows.

In the present study, high doses of acetylsalicylic acid caused changes in the neurochemical phenotype of nerve cells. Immunofluorescence staining showed the presence of all studied neuroactive substances in all plexuses of the porcine jejunum (Figure 1). Under physiological conditions, the highest population of neurons in the myenteric plexus were nerve cells immunoreactive (IR) to nNOS. In control animals, the proportion of nNOS-positive neurons in the MP was 32.3% ± 1.25 of MP (Figure 2A–C), while VIP was 10.28% ± 0.28 (Figure 2G–I) and CART was 9.53% ± 0.15 (Figure 2M–O). In aspirin-treated piglets, the percentage of nNOS-IR myenteric neurons decreased (21.34% ± 0.5) (Figure 2D–F), but the percentages of VIP-IR (13.14% ± 0.55) (Figure 2J–L) and CART-IR (14.04% ± 0.11) (Figure 2P–R) were significantly increased when compared with controls.

**Figure 3:**
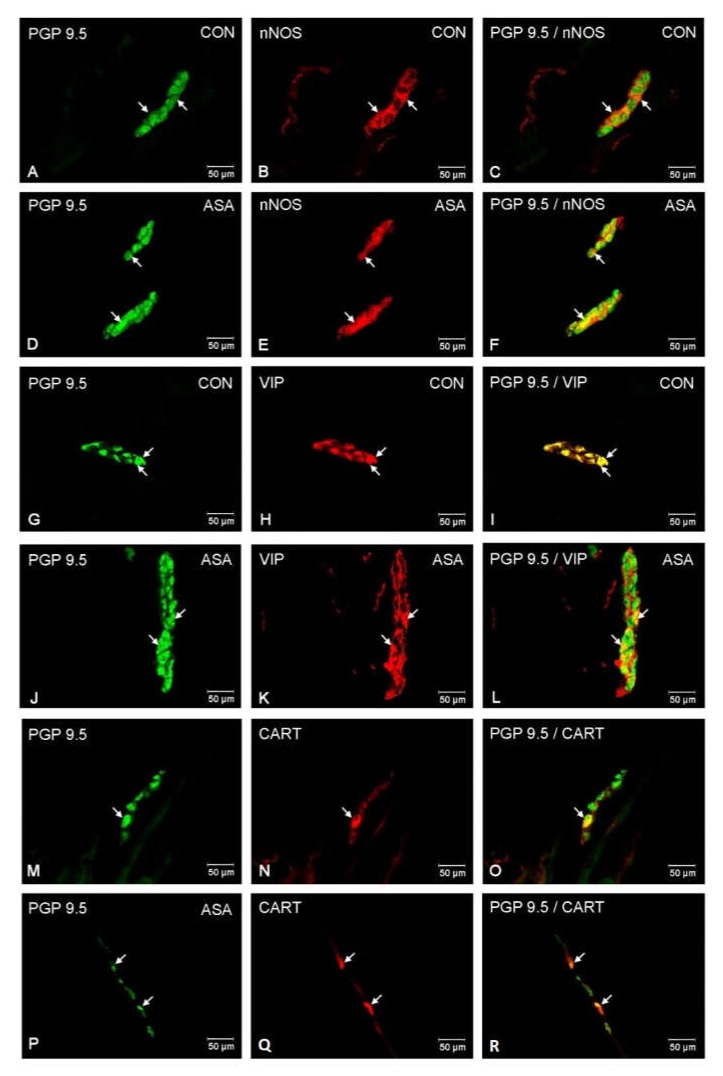
Neuron bodies within outer submucous plexuses of porcine jejunum containing the protein gene-product 9.5 (PGP 9.5) (used as pan neuronal marker), neuronal nitric oxide synthase (nNOS), vasoactive intestinal peptide (VIP) and cocaine- and amphetamine- regulated transcript peptide (CART) under physiological condition (CON) (**A**–**C**,**G**–**I**,**M**–**O**) and after aspirin supplementation (ASA) (**D**–**F**,**J**–**L**,**P**–**R**). The right column of the pictures (**C**,**F**,**I**,**L**,**O**,**R**) has been created by digital superimposition of two colour channels, and colocalization of both antigens in the studied neuron bodies was indicated with arrows.

**Figure 4:**
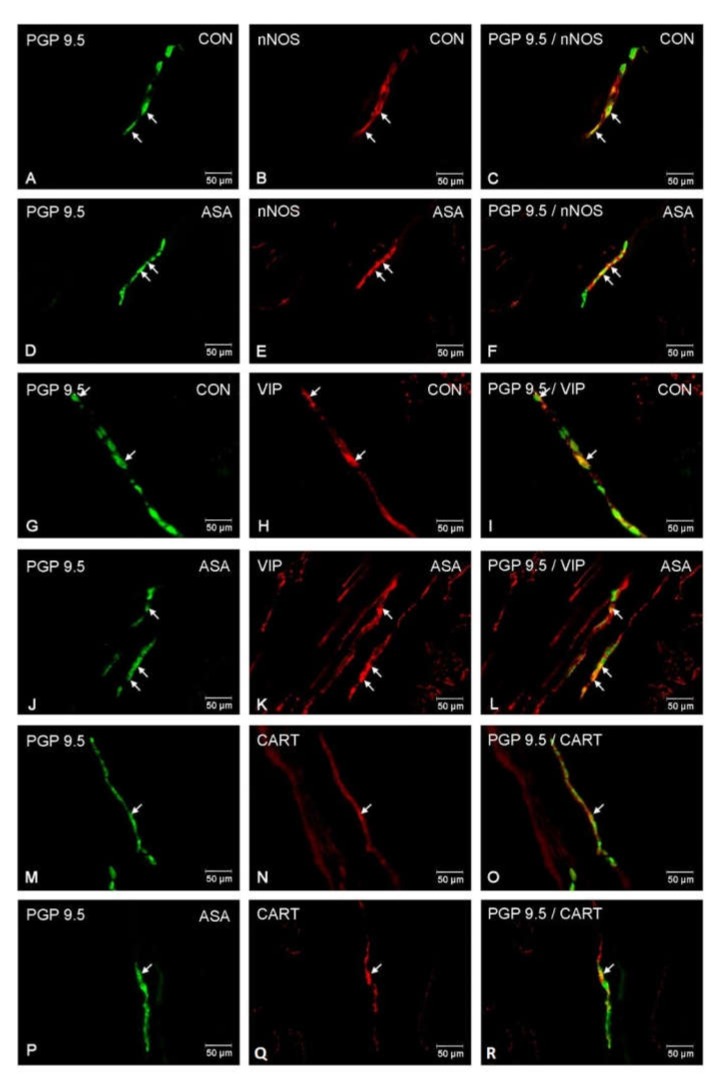
Neuron bodies within inner submucous plexuses of porcine jejunum containing the protein gene-product 9.5 (PGP 9.5) (used as pan neuronal marker), neuronal nitric oxide synthase (nNOS), vasoactive intestinal peptide (VIP) and cocaine- and amphetamine- regulated transcript peptide (CART) under physiological condition (CON) (**A**–**C**,**G**–**I**,**M**–**O**) and after aspirin supplementation (ASA) (**D**–**F**,**J**–**L**,**P**–**R**). The right column of the pictures (**C**,**F**,**I**,**L**,**O**,**R**) has been created by digital superimposition of two colour channels, and colocalization of both antigens in the studied neuron bodies was indicated with arrows.

In both control and experimental groups, the proportion of the studied substances in the submucous plexuses (outer and inner) were altered (Figure 1B,C). After aspirin treatment, the expression of nNOS in submucous neurons was significantly decreased to 4.42% ± 0.16 in the OSP (Figure 3D–F) in comparison to 9.6% ± 0.23 for controls (Figure 3A–C) and 3.09% ± 0.2 in the ISP (Figure 4D–F) in comparison to 6.77% ± 0.16 for controls (Figure 4A–C). In control animals, the presence of VIP was detected in 13.98% ± 0.39 of outer submucous neurons (Figure 3G–I) as well as 16.73% ± 0.41 of inner submucous neurons (Figure 4G–I). Aspirin administration distinctly increased the proportion of VIP-IR outer submucous neurons to 20.49% ± 0.19 (Figure 3J–L) and inner submucous neurons to 21.44% ± 0.3 (Figure 4J–L). In both control and experimental animals, CART-positive submucous nerve cells were occasionally found. The percentage of CART-positive neurons in the OSP was 2.89% ± 0.15 in control (Figure 3M–O) and 4.09% ± 0.22 in experimental animals (Figure 3P–R), while in the ISP of control and experimental animals it was 2.39% ± 0.12 (Figure 4M–O) and 3.34% ± 0.10 (Figure 4P–R), respectively.

## 3. Discussion

The present immunohistochemical studies revealed that in the jejunum of pigs treated with high doses of acetylsalicylic acid subpopulations of NOS-, VIP- and CART-IR myenteric and submucous neurons were statistically altered. Previous studies have also shown changes in these neuroactive substances in the swine gastrointestinal tract during induced inflammatory processes [3,20,24]. It is believed that the capacity to change neuropeptide/neurotransmitter content, termed enteric neuroplasticity, is an adaptation to an unfavorable enteric microenvironment [29]. This study confirmed that long-term ASA supplementation induces changes in the porcine jejunum, and that ENS responds to this harmful condition with neuronal plasticity.

One of the most important neurotransmitters is nitric oxide (NO) [24]. At least three isoforms of nitric oxide synthase (NOS) have the possibility of intracellular synthesis of nitric oxide from L-arginine [30,31]. In the GIT, under physiological conditions, NO is primarily produced by the constitutive neuronal form of NOS (nNOS) and is released from inhibitory neurons of the enteric nervous system [32]. The function of nitric oxide in the digestive tract depends on the site of production. NO synthesized in myenteric and submucous neurons is the main non-adrenergic, non-cholinergic (NANC) inhibitory neurotransmitter that mediates smooth muscle relaxation in the GIT and is therefore an important factor involved in gastric and intestinal contractility [33,34]. Moreover, in the enteric nervous system, NO is involved in the regulation of myenteric and intestinal blood flow by acting as a vasodilator [35], reducing the secretion of electrolytes and intestinal hormones [21,34], as well as maintaining mucosal integrity of GIT [34]. Finally, nitric oxide is associated with changes in nNOS immunoreactivity in intestinal nerves, which is especially prominent in pathological processes, such as during inflammation [21], Crohn’s disease [36] and mycotoxin poisoning [37].

In this study, we have shown that nNOS occurs in relatively high proportion of enteric neurons, which is in agreement with previous studies, where nitrergic structures have been noted in different mammalian species such as pigs [20,38], guinea pigs [39], mice [39,40] rats [41,42] and humans [43]. The current findings are congruent with the available literature, where more numerous NOS-positive neurons were found in the myenteric plexus [22,44]. Less than 10 % of neurons in both submucous plexuses (outer and inner) were immunoreactive to nNOS, which is similar to previous studies conducted in pigs [22]. To date, there are only a few studies focusing on the distribution of nNOS in the porcine ENS, particularly in the jejunum. Most studies assess changes in the expression of nNOS in the stomach [45,46], while modifications in the small intestine are poorly described. Our data showing the relatively large population of nNOS-positive enteric neurons confirms the previously described roles of NO in the regulation of physiological processes within the GIT, as well as during pathological stages.

During our study, a clear influence of supplementation of aspirin on nNOS-IR neurons was observed in the jejunum. In all plexuses, we observed downregulation of nNOS expression in intramural neurons. The current results show that high doses of aspirin administration contribute to a decrease in the proportion of nNOS-positive nerve structures, particularly in the myenteric plexus. In turn, the populations of nNOS-expressing nerve cells in both the outer and the inner submucous plexuses were also significantly decreased but to a lesser extent than in the MP. Reduction of nNOS-IR enteric neurons was previously observed during diabetes [44], Chagas’ disease [45], Hirschsprung’s disease [46], as well as ischemia [22]. In contrast, an increase in the proportions of nNOS-IR enteric intramural neurons was found in the swine small intestine during proliferative enteropathy [47], ileitis [22], inflammatory bowel disease (IBD) [48]. Consistent changes in nNOS expression in various disease models may implicate dysregulation of this enzyme in local tissue injury. It is well known, that prostaglandins and NO play a key role in defending the gastrointestinal mucosa, and this phenomenon is termed “gastric mucosal defense” [49]. The important role of these mediators is demonstrated by the fact that suppression of NO synthesis or mucosal prostaglandins contributes to the higher susceptibility of the GIT to injuries [49,50]. In addition to ASA being a well-known negative factor affecting the intestinal mucosa, the present study shows that acetylsalicylic acid can be an agent that changes the chemical phenotype of nitrogenic neurons supplying the jejunum.

Previous studies have shown neuroprotective properties of NO in the enteric nervous system, and data on the neurochemical adaptation of the ENS are well established [44]. The main regulator of nNOS activity is free cytosolic Ca^2+^, which stimulates nNOS during its interaction with calmodulin. Arrival of action potentials activates voltage-dependent Ca^2+^ channels located in the neurolemma and stimulates the release of Ca^2+^ from intracellular stores that elevates cytosolic Ca^2+^ concentrations [32]. Based on the present results indicating a decrease in expression of nNOS, we can assume that this is an adaptation to the induced inflammatory stage. Although a variety of factors can contribute to the propensity of NOS neurons to be involved in enteric neuropathies, the most important seems to be a defect in neurons to maintain Ca^2+^ homeostasis. Generally, in neurons, stress associated with inflammation can increase cytoplasmic Ca^2+^ and as a consequence cause Ca^2+^-toxicity. This may lead to overproduction of NO, whose free radical properties may cause cell damage, which is exacerbated by peroxynitrite formed when NO reacts with oxygen free radicals [51]. We can assume that in order to avoid such a situation and the resulting side effects, the intestinal nervous system intervenes by reducing expression of nNOS, a potentially adaptive process in the ENS.

Another biologically active substance present in the ENS is vasoactive intestinal polypeptide (VIP). VIP is considered to be one of the most important neuropeptides involved in intestinal processes [3]. First of all, VIP acts as an inhibitory agent causing the smooth muscles relaxation [52,53], inhibition of motor activity of the stomach [2], as well as suppression of the gastric acid secretion. Moreover, VIP participates in the stimulation of mucus secretion, increases the production of intestinal juice and bile and takes part in blood flow regulation [54]. Furthermore, VIP is known as a key regulator of immunological processes and is considered to be one of the main neuroprotective factors within the ENS [28,55].

In the present study, VIP-IR nervous structures were observed within all plexuses investigated in both control and experimental animals. The obtained results are consistent with previous findings, where porcine VIP has been isolated from the small intestine of pig [28]. Moreover, VIP was found in all types of intramural plexuses in the rat [56], guinea pig [57], golden hamster [58] and human [59]. The presence of VIP-positive neurons confirms the previously described important functions of this neuroactive substance in intestinal processes. In studies conducted on gold hamster tissues, only a small number of VIP-IR neurons were described in ileum, colon and caecum [58]. On the other hand, in the porcine duodenum, the percentage of VIP-IR neurons was 11.90 ± 0.30% in MP, 13.78 ± 1.07% in OSP and 17.63 ± 0.77% in ISP [26]. This is in line with our observations. According to the available literature, there are discrepancies in the number of neurons immunoreactive to VIP in different regions of GIT, as well as between animal species. This diversity may suggest that the precise functions of VIP in GI regulatory processes depend on the segment of the alimentary tract.

Our findings of increased expression of VIP in the jejunum intramural neurons as a consequence of aspirin administration provides convincing evidence of the important role of this peptide in neuronal responses this NSAIDs treatment. Moreover, it is well established that under the influence of multiple pathological stimuli, an overexpression is usually noted in the case of substance that promotes the regeneration of nerve cells [60]. Otherwise, it has been shown that the pig is a good animal model for a VIP anti-inflammatory preclinical study. Evidence of increased expression of VIP following inflammation was found in inflammatory bowel disease (IBD), ulcerative colitis [61], Crohn’s disease [62,63], gastric ulcer [2], diabetes [44], dextran sodium sulphate-induced colitis in rats [64], guinea-pig TNBS colitis [65], *Helicobacter pylori* infection [66], and axotomy [19]. Generally, VIP anti-inflammatory and neuroprotective properties are achieved by down-regulation of pro-inflammatory and synthesis of ant-inflammatory cytokines [22]. Based on our obtained results, we can conclude that neurotrophic and neuroprotective actions are among the major roles of VIP in the ENS. Furthermore, VIP and nNOS are often coexpressed in neurons of the ENS [67], which agrees with our present study. According to Bulc et al. [44], an increase in VIP-immunoreactivity accompanied by a decrease in nNOS expression can protect nerve cells from neuronal death.

Cocaine- and amphetamine-regulated peptide transcript (CART) is a relatively recently discovered substance, which acts as a neuromodulator of nerve structures in the GIT. CART was first observed in 1981 in the ovine hypothalamus and has since been described in different fragments of the GIT of numerous mammals. Available literature indicates the presence of this active substance in various species, including pig [54], human [68], and rat [69]. The exact role of CART in gut function is still unknown, but the localization of CART-positive neurons within the ENS may indicate its properties. Bulc et al. revealed that in human cecum, CART-IR nerve fibers also show a high degree of co-localization relative to VIP and nNOS [67].

CART was found in numerous myenteric nerve cells throughout the jejunum, while CART-expression in both submucous plexuses was scarce. The large population of CART-IR neurons in the MP may suggest that this active substance is involved in the regulation of intestinal motility. Other authors have also observed a high number of myenteric CART-positive nerve cells in the porcine small intestine, which is consistent with results obtained in our experiment [23,70]. Possible motor functions of CART were also studied in vitro in rats (stomach, ileum and colon) and a similar dependence was found [69]. In our study, only a few scattered CART-expressing neurons could be detected in the inner and outer submucous ganglia. Similar results were obtained by Wojtkiewicz et al. [6], where the number of CART-IR neurons in the OSP of the porcine jejunum amounted to 2.7% ± 2.2. Interestingly, a previous study assessing the stomach has shown that there are no CART-like immunoreactive cell bodies in the gastric submucous ganglia [71]. Moreover, some authors suggest that CART participates in inhibition of feeding [54], reduction of gastric acid secretion [72], the stress response [24], and exacerbation of colonic motility [73].

This is the first study demonstrating the changes in the immunohistochemical characteristics of CART in intramural neurons of the porcine jejunum following aspirin intoxication. During our study, the evident influence of induced inflammatory stage on CART-IR neurons has been observed in studied segment of GIT. The present study confirms and extends previous observations that CART is overexpressed during intestinal inflammation and suggests it may play a role in neuroprotection [73,74]. This is consistent with the findings of previous studies, where enhanced expression of CART was observed during numerous pathological processes [23], such as mycotoxin intoxication [75] and hypertension in rats [76]. Oppositely, the down-expression of CART has been noted in cases of Hirschsprung’s disease [23] and diabetes mellitus [68]. We can speculate that dysregulation of CART expression probably depends on both the pathological condition and the region of the GIT studied. Moreover, it is well established that CART in enteric neurons of the porcine GIT [54,77], as well as human [68], can colocalize with a wide range of other neurotransmitters. The coexistence of CART and nNOS or VIP in the same nervous structures can suggest a similar role of colocalized neuropeptides. Thus, CART may be implicated in promoting the survival of porcine enteric neurons, as well as in the protection of ENS nerve cells in intestinal disorders, injuries or neuronal stress. However, further studies are needed to explain the exact physiological function of CART within the porcine GIT.

## 4. Materials and Methods

All experimental procedures were approved by the Local Committee for Animal Experiments at the University of Warmia and Mazury in Olsztyn (Permit Number: 54-2017 from the 25th July 2017) and carried out in accordance with its recommendations. The study was performed on approximately 8-week old Pietrain × Duroc immature gilts (*n* = 8) weighing approximately 20 kg. The animals were divided into 2 groups—control (*n* = 4) and experimental (*n* = 4). The control gilts received empty gelatin capsules orally over a period of 28 days, while pigs from the experimental group were given acetylsalicylic acid (ASA) orally (Aspirin, Bayer, Leverkusen, Germany, 100 mg/kg of body weight). All animals were housed in the breeding pens in standard laboratory conditions with free access to fresh water (ad libitum) and feed mixture appropriate to the age. The temperature in the animal pens was maintained at 21 °C and the humidity was within 50% to 60%. The experiment lasted 4 weeks. After this period, pigs were preanesthetized with azaperone (Stresnil, Janssen Pharmaceutica N.V., Beerse, Belgium, 2 mg/kg of body weight intramuscularly). Animals were euthanized by an overdose with sodium pentobarbital (Motbital, Biowet Puławy Sp. z o.o, Puławy, Poland, intravenously). After confirming the cessation of biological functions, tissue was collected for further research. Fragments of jejunum (2 cm long) located approximately 45 cm from the pylorus were collected from all pigs. The material was fixed by immersion in 4% (*v*/*v*) buffered paraformaldehyde (pH 7.4) for 2 h, washed 3 times in 0.1 M phosphate buffer (pH 7.4) with 24 h exchange of the buffer and transferred into 18% (*v*/*v*) buffered sucrose solution (pH 7.4). Tissue was stored at 4 °C for 2 weeks. After this period, the collected intestinal fragments were embedded in OCT Tissue-Tek and cut at −22 °C into 14 μm thick sections using a cryostat (Microm, type HM525, Walldorf, Germany) and placed on degreased microscope slides.

The frozen sections of jejunum were subjected to labeling using routine double-immunohistochemical staining. Briefly, slides were dried at room temperature for 30 min. Subsequently, preparations were washed in a 0.1 M phosphate-buffered saline solution (PBS, pH 7.4, 3 × 15 min), followed by incubation in a humidified chamber with blocking solution composed of 10% (*v*/*v*) normal goat serum, 0.1% (*v*/*v*) bovine serum albumin in 0.1 M PBS, 0.25% (*v*/*v*) Triton X-100, 0.01 % (*v*/*v*) NaN_3_ and 0.05 % (*v*/*v*) Thimerosal (1 h, room temperature). After washing three times in PBS (15 min), the fragments of jejunum were incubated at room temperature overnight with a mixture of primary antibodies against protein gene-product 9.5 (a pan neuronal marker), nitric oxide synthase (nNOS), vasoactive intestinal peptide (VIP) and cocaine- and amphetamine-regulated transcript peptide (CART). The following day, slides were washed with PBS (3 × 15 min) and incubated for 1 h at room temperature with the appropriate secondary antibodies (Alexa Fluor 488 and Alexa Flour 546). Table 1 presents the specifications of the used antibodies. Slides were washed with PBS (3 × 15 min), coverslipped using a solution of glycerol and carbonate buffer (pH 8.4) and stored at 4 °C until ready for use. To verify specificity of immunohistochemical labeling, the typical specificity test controls were performed (standard controls, i.e., pre-absorption for the neuropeptide antisera with appropriate antigen, omission, as well as replacement of primary antisera by non-immune sera).

Stained sections of the jejunum were examined under an Olympus BX51 microscope equipped with epifluorescence and appropriate filter sets. Photographic documentation was made by using a digital camera connected to a PC and processed with Olympus Cell F image analysis software (Olympus, Tokyo, Japan). The percentage of neurons was determined by counting cells showing co-localization of two substances (PGP 9.5 and one of the tested substances) in relation to the total number of neurons in a given population. Thus, to calculate the proportion of VIP-positive neurons (analogously nNOS- and CART-positive) in the jejunum from each type of ganglia (muscular, submucous external and internal), the number of neurons immunoreactive to VIP (or nNOS or CART) was expressed as a percentage of the total number of PGP 9.5-positive neurons. A total number of 500 PGP 9.5 neurons per animal were counted. In this study, the total number of neurons immunoreactive to PGP 9.5 was considered as 100%. In order to avoid double counting of the same neurons, the principle was adopted that further sections stained with the same combination of antibodies were at least 200 μm away from each other. Only nerve cells that had a visible cell nucleus were considered, pooled and presented as a mean ± standard error of mean (SEM).

The results from each group (control and experimental) were analyzed statistically with Statistica 12 software (StatSoft Inc., Tulsa, OK, USA). Significant differences were assessed using the Student’s *t*-test for independent samples (* *p* < 0.05, ** *p* < 0.01, *** *p* < 0.001).

## 5. Conclusions

Long-term supplementation of high doses of aspirin (100 mg/kg/day per 28 days) causes significant changes in the expression of neurotransmitters in the enteric neurons of the porcine jejunum. It is a widely recognized dose used in experiments aimed at understanding the effects of acetylsalicylic acid on gastrointestinal function. A similar dose was used in pigs [16], rats [78], rabbits [79] and mice [80]. High dose administration aims to provide a research model for people abusing aspirin as well as for patients receiving long-term acetylsalicylic acid as a result of cardiovascular disorders.

In the peripheral nervous system, including ENS, plasticity revealed by modification of the expression of many neurotransmitters is common in response to nerve damage as well as drugs treatment or the administration of toxic substances. The obtained results showed that enteric neurons are adaptable to changed conditions.

## Figures and Tables

**Table 1:** Antibodies used for immunochemistry.

Primary Antibodies				
**Antigen**	**Code**	**Host Species**	**Dilution**	**Supplier**
PGP 9.5	7863-2004	Mouse	1:1000	Bio-Rad, Hercules, CA, USA
nNOS	AB5380	Rabbit	1:3000	Sigma-Aldrich, Saint Louis, MO, USA
VIP	AB22736	Rabbit	1:2000	Abcam, Cambridge, UK
CART	H-003-61	Rabbit	1:16000	Phoenix Pharmaceuticals, Inc., Burlingame, CA, USA
**Secondary Antibodies**				
**Reagent**	**Code**		**Dilution**	**Supplier**
Alexa Fluor 488 nm donkey anti-mouse IgG	A21202		1:1000	ThermoFisher Scientific, Waltham, MA, USA
Alexa Fluor 546 nm goat anti-rabbit IgG	A11010		1:1000	ThermoFisher Scientific, Waltham, MA, USA
